# Molecular and Biological Characterization of Chinese Sacbrood Virus LN Isolate

**DOI:** 10.1155/2011/409386

**Published:** 2011-03-03

**Authors:** Ma Mingxiao, Li Ming, Cheng Jian, Yang Song, Wang Shude, Li Pengfei

**Affiliations:** Laboratory Animal Center, Liaoning Medical University, Jinzhou 121001, China

## Abstract

Chinese sacbrood virus (CSBV) was purified from diseased insects, and its genome was cloned and sequenced. The genomic RNA of CSBV is 8863 nucleotides in length and contains a single large open reading frame encoding a 319.614 kDa polyprotein. The coding sequence is flanked by a 178-nucleotide 5′ nontranslated leader sequence and a 142-nucleotide 3′ nontranslated region, followed a poly(A) tail. Four major structural proteins, VP1,VP2, VP3 and VP4, were predicted in the N-teminal of the polyprotein. The C-terminal part of the polyprotein contains sequence motifs which is a typical and well-characterized picornavirus nonstructural proteins: an RNA helicase, a chymotrypsin-like 3C protease, and an RNA-dependent RNA polymerase. Genetic analysis shows that the CSBV-LN had a 13-amino-acid deletion at amino acid positions 710–719 and 727–729 in comparison with CSBV-GZ and SBV-UK, and the SBV-UK had a 7-amino-acid deletion at amino acid positions 2124–2132 in comparison with CSBV-GZ and CSBV-LN, and the CSBV-GZ and CSBV-LN had a 6-amino-acid deletion at amino acid positions 2143–2150 in comparison with SBV-UK. Phylogenetic analysis using RdRp of selected picorna-like viruses shows that CSBV/SBV and Deformed Wing Virus (DWV) tend to group together, which possesses an RNA of similar size and gene order.

## 1. Introduction

The scientific interest in viral diseases of the honeybee (Apis mellifera L.) has been increasing considerably during the past few years [1]. At least 18 different viruses have been detected in honeybees so far. Although usually not associated with clinical symptoms, viruses in certain cases may cause serious or even lethal disease in individual bees or the collapse of entire colonies.

Sacbrood virus (SBV) primarily affects the brood of the honeybee and results in larval death [2]. Infected larvae fail to pupate, and ecdysial fluid aggregates around the integument, forming the “sac” for which the disease is named. Infected larvae change in color from pearly white to pale yellow, and shortly after death they dry out, forming a dark brown gondola-shaped scale [3]. SBV may also affect the adult bee, but in this case obvious signs of disease are lacking [4, 5]. Such bees may, however, have a decreased lifespan [5, 6]. Sacbrood occurs most frequently in spring, when the colony is growing most rapidly and large numbers of susceptible larvae and young adults are available [5]. 

SBV infecting Chinese honeybee was named Chinese sacbrood virus (CSBV). CSBV was first described in Guangdong China in 1972 and reemerged in Liaoning China in 2008, which caused lethal disease in individual bees or the collapse of entire colonies.

Chinese sacbrood virus (CSBV) is 26–30 nm in diameter, nonenveloped, round, and featureless in appearance and belongs to the small RNA virus family, the *Picornaviridae*. The genome of the virus is +ssRNA, and there are four structural proteins (30.5, 31.5, 37.8 kDa, and 44.2 kDa) at its capsid [7], but three structural proteins (25, 28, and 31.5 kDa) have been reported about SBV [8, 9]. A small VP4-like protein has not been detected [10]. SBV is the first honeybee virus which has been completely sequenced and its genomic RNA is longer (8,832 nucleotides (nt)) than that of typical mammalian picornaviruses (approximately 7,500 nt) and contains a single, large open reading frame encoding a polyprotein of 2,858 amino acids (Genbank accession no. AF092924.1). But The CSBV genome sequence was 8740 nucleotides except the 3′ and 5′ ends, which contains a single, large open reading frame encoding a polyprotein of 2,861 amino acids (Genbank accession no. AF469603). The genomic organization of SBV/CSBV clearly resembles that of typical members of the Picornaviridae, with structural genes at the 5′ end and nonstructural genes at the 3′ end arranged in a similar order. 

However we recently characterized a CSBV from Liaoning China. The Liaoning CSBV will be referred to as CSBV-LN to distinguish it from the original isolates, which will be referred to as CSBV-GZ (Genbank accession no. AF469603) and SBV-UK (Genbank accession no. AF092924.1) according to the phylogenetic analysis and comparison of characteristics.

## 2. Materials and Methods

### 2.1. Samples Purification

CSBV(CSBV-LN) was obtained from a natural outbreak in Liaoning China. Infected larvae/pupae were stored frozen at −70°C until required. For purification of the virus, CSBV-infected larvae were homogenized in 5 mL NT buffer (100 mM NaCl, 10 mM Tris, pH 7.4) and the macerate was clarified at 1000 g for 10 min. The supernatant was extracted with an equal volume of 1,1,2-trichlorotrifluoroethane before the aqueous phase was layered over a discontinuous CsCl gradient (1.5 g/cm^3^ and 1.2 g/cm^3^) and centrifuged at 270000 g for 1 h in an SW50 rotor. The material at the CsCl interface was harvested and adjusted to a volume of 5 mL (final density 1.38 g/cm^3^) with CsCl solution and centrifuged at 270000 g overnight. Two light-scattering bands were formed which were collected separately and diluted with NT buffer, and the virus was collected by sedimentation at 270000 g for 1 h. Pellets were resuspended in NT buffer. 

### 2.2. Electron Microscopy and SDS-PAGE

Samples taken throughout the virus purification procedure were cleared by low-speed centrifugation (10 min at 8,000 g), and 100 *μ*L of supernatant was pelleted directly onto carbon-coated Formvar copper grids by ultracentrifugation (15 min at 82,000 g) with a Beckman Airfuge. The grids were negatively stained with 2% sodium phosphotungstate at pH 6.8 for 90 seconds and observed with a Philips CM10 transmission electron microscope. 

**Figure 1:**
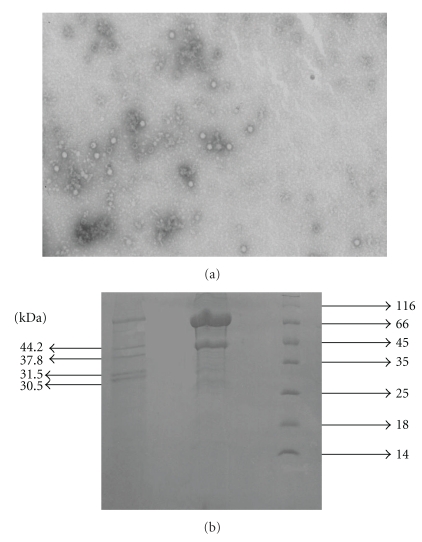
(a) Observed virus particles of CSBV by electron microscopy. (b) The SDS-PAGE of CSBV (1) Structural proteins (2) Negative control (3) Protein Marke.

The four main structural proteins of CSBV were separated by SDS-PAGE (Figure 1). Structural proteins were resolved on 12% SDS-PAGE gels by using standard protocols [12]. Proteins were blotted onto PVDF membranes.

### 2.3. Amplification of the Full-Length CSBV-LN Genome

Primers used in this work (Table 1) were designed on the basis of the nucleotide sequence of CSBV-GZ (Genbank accession no. AF469603) and SBV-UK (Genbank accession no. AF092924.1). They were designed for the production of full-length single-stranded cDNA of the CSBV-LN genome. S1–S9 primer pairs were selected based on the CSBV-GZ sequence, and S10 primer pair was selected based on the SBV-UK genome.

Intact viral genomic RNA was extracted from the purified virus preparations by TRIzol-LS (Invitrogen) and retrotranscribed into cDNA by using AMV reverse transcriptase and random oligonucleotides and oligo (dT) [13] as primer. The cDNA was amplified by PCR for 30 cycles, with annealing at 50 to 55°C for 30 seconds and elongation at 72°C for 50–60 seconds, depending on the primer sets in Table 1. The 3′ end of the CSBV-LN genome was cloned by the 3′-RACE technique (Clontech). The PCR amplification of this genome was cloned into the pMD-18-T vector (TaKaRa Biotechnology (Dalian) Co., Ltd).

### 2.4. Sequence and Phylogenetic Analyses

Nucleotide sequences were resolved on an ALI377 automated DNA sequencer in TaKaRa Biotechnology (Dalian) Co., Ltd and assembled with Lasergene (DNASTAR), and the putative protease cleavage sites were predicted using NetPicoRNA 1.0 Server from http://www.cbs.dtu.dk/services/NetPicoRNA/ [14].

## 3. Results

### 3.1. Virus Identification and Molecular Characterization

Electron microscopy revealed the presence of large amounts of empty and filled icosahedral virus particles of about 26 nm in diameter in virus preparations from infected larvae/pupae (Figure 1(a)) that were absent in control preparations from healthy bees.

The purified virus particle has four major proteins, with apparent masses of 30.5, 31.5, 37.8, and 44.2 kDa (Figure 1(b)). The results are similar to those of CSBV-GZ [7].

### 3.2. Nucleotide Sequence Analysis

**Figure 2:**
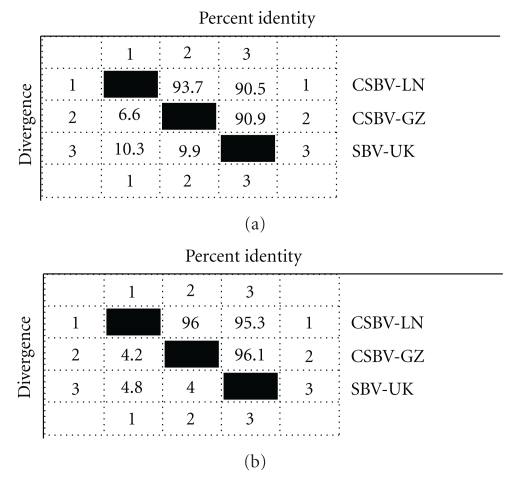
The CSBV-LN was compared with CSBV-GZ and SBV-UK. The homologies of ORF sequences are 93.7% and 90.5% to SBV-UK, respectively (a). The CSBV-LN was compared with CSBV-GZ and SBV-UK. The homologies of deduced amino acid are 96% and 95.3%, respectively (b).

The complete viral genome sequence is 8863 nucleotides long and is enriched in A (30.06%) and U (29.52%) compared to G (24.40%) and C (16.02%). The genome contains one large open reading frame (ORF) encoding 2848 amino acids and commencing at nucleotide 178. There is a second in-frame AUG codon at 418, but AUG 178 may be the translation initiation site since unlike AUG 418 it occurs in a context (AUUAUGG) identical to that of many invertebrate initiating codons (ANNAUGG). But the second in-frame AUG codon position of CSBV-LN (at nucleotide 418) is different from that of SBV-UK, whose second in-frame AUG codon is at 197 [10]. The ORF ends with a UAG stop codon at nucleotide 8721, encoding a product of 319613.75 Daltons. The CSBV 3′ untranslated region (UTR) (79 nucleotides) is of a similar size to those of mammalian picornaviruses (40–126 nucleotides). The 5′ UTR was produced from CSBV-LN by RT-PCR with the primer on the basis of SBV-UK (Genbank accession no. AF092924.1), so the CSBV 5′ UTR is similar to SBV-UK (177 nucleotides). The CSBV-LN ORF sequences and deduced amino acid are 93.7% and 96% identical to GSBV-GZ, respectively, and 90.5% and 95.3% identical to SBV-UK, respectively (Figure 2). 

### 3.3. Amino Acid Sequence Analysis

**Figure 3:**
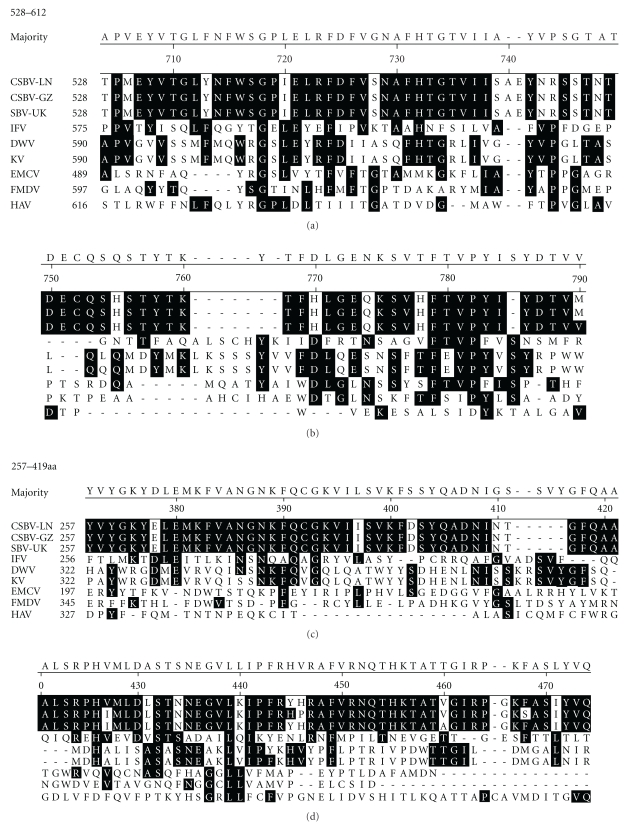

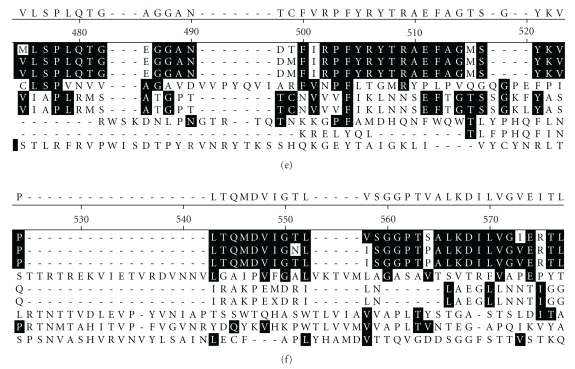
Alignment of residues 528–612 and 257–419 of CSBV-LN was compared with CSBV-GZ and SBV-UK. IFV: infections flacherie virus; DWV: deformed wing virus; EMCV: encephalomyocarditiss; HAV: hepatitis A virus; FMDV: foot and mouth disease virus.

**Figure 4:**
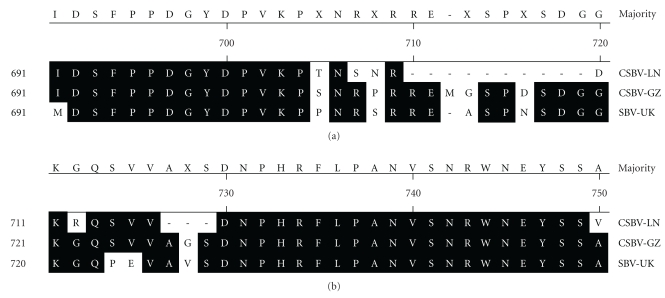
In comparison with CSBV-GZ and SBV-UK, the CSBV-LN had a 13-amino acid deletion at amino acid positions 710–719 and 727–729.

Domain analysis of the polyprotein shows the order of the viral proteins to be identical to that of mammalian picornaviruses [15], with the structural proteins in the N-terminal portion and the nonstructural proteins in the C-terminal portion of the polyprotein. The amino acid sequence deduced from the N-terminus of the CSBV polyprotein was aligned with structural genes from mammalian picornaviruses and insect picorna-like virus. The alignment (Figure 3) showed similar result with SBV-UK [9], whose residues 528–612 of CSBV exhibited similarity to the structural proteins of VP3 of mammalian picornaviruses foot and mouth disease virus (FMDV, Genbank accession no. AY333431.1), hepatitis A virus (HAV, Genbank accession no. AB279735), and encephalomyocarditiss (EMCV, Genbank accession no. M81861.1). However, this region has been designated previously as VP1 of infections flacherie virus (IFV, Genbank accession no. AB000906). A second region identity was found between residues 257 and 419 of CSBV, which resembled VP2-like proteins of FMDV, EMCV Kakugo virus (KV, Genbank accession no. AB070959), and deformed wing virus (DWV, Genbank accession no. AJ489744), the VP3 region of IFV. But in comparison with CSBV-GZ and SBV-UK, the CSBV-LN had a 13-amino acid deletion at amino acid positions 710–719 and 727–729 (Figure 4). The amino acid composition for the protein sequences was analysed using ProtParam tool from http://expasy.org/tools/protparam.html/ [16].

**Figure 5:**
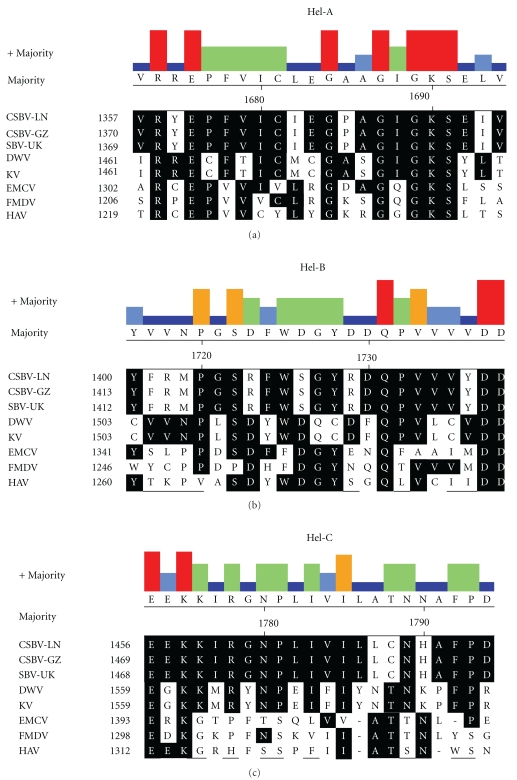
Alignment of putative CSBV RNA helicase domain: alignment of the conserved regions of the putative RNA helicase region from CSBV-LN, CSBV-GZ, SBV-UK, DWV, KV, IFV, EMCV, HAV, and FMDV. The motifs identified by Koonin and Dolja [11] are labelled A, B, and C. The numbers on the left show the amino acid position of the aligned sequences. A histogram of agreement between the majority consensus and each column of aligned residues is displayed. Strong agreement is indicated by height and color, with red being the strongest.

The helicase domains A, B, and C [11] are found between amino acids 1357 and 1477 (Figure 5), including the perfectly conserved putative nucleoside triphosphate-binding residues ^1368^GxxGxGKS^1378^ and ^1415^QX_5_DD^1422^ in domains A and B, but the C domain appears least well conserved, containing only three of six residues potentially associated with the site. 

The 3C protease domains span amino acids 2146 to 2277 and conserve the cysteine protease motif ^2254^GxCG^2257^ and the putative substrate binding residue ^2271^GxHxxG ^2276^(GMHFAG). C^2256^ [17] is the third residue of the protease catalytic triad that also involves a histidine residue and either an aspartate or glutamate residue [18, 19]. ^2153^H and E^2193^ are the most likely candidates between the processing site at position 2133 and the cysteine protease motif to complete the catalytic triad with C^2256^. 

**Figure 6:**
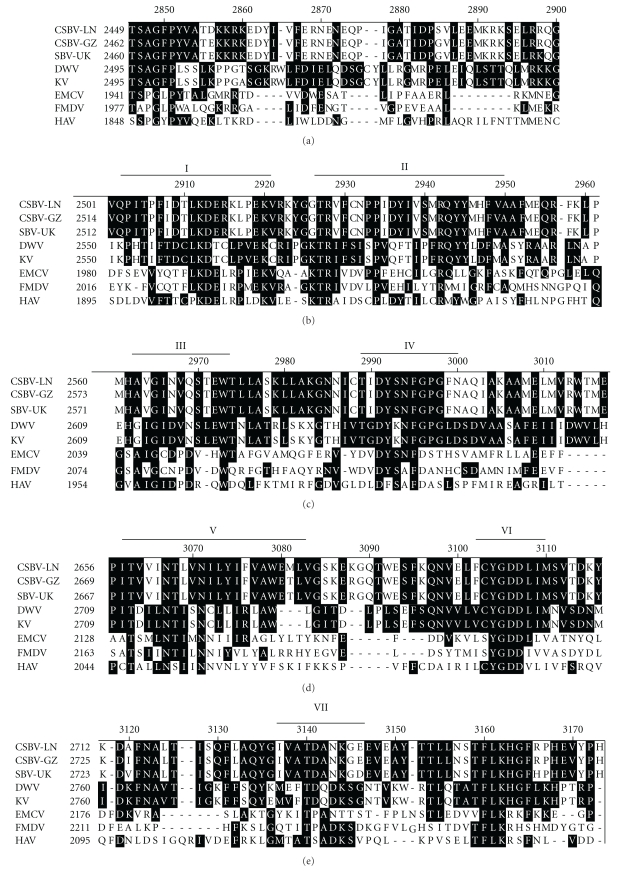

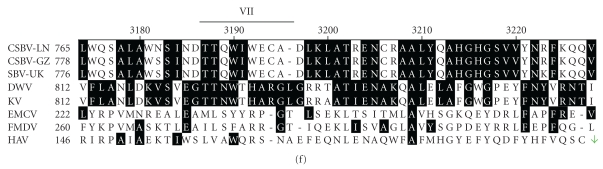
Alignment of the putative RdRp domain with those of other viruses. Residues identical in at least nine viruses are shown in inverse typeface. The motifs identified by Koonin and Dolja [11] are labeled I–VIII. The numbers on the left show the amino acid position of the aligned sequences.

The C-terminal region (amino acids 2449–2818) of the CSBV polyprotein was similar to sequences of RdRp of viruses of the Picornaviridae (Figure 6). All eight features conserved in RdRp of positive-strand RNA viruses [11] were also identified in this region of the CSBV genome, with only one exception: motif IV contains a glycine (amino acid 2595) whereas the consensus indicates that this residue is aspartic acid.

**Figure 7:**
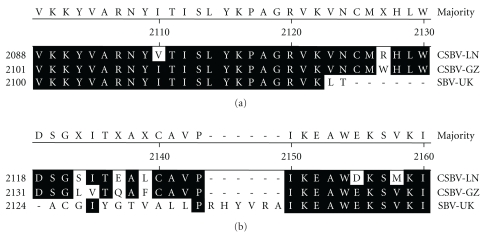
The SBV-UK had a 7-amino-acid deletion at amino acid position 2125–2131 in Comparison with CSBV-GZ and CSBV-LN, and the CSBV-GZ and CSBV-LN had a 6-amino-acid deletion at amino acid position 2144–2149 in Comparison with SBV-UK.

However the SBV-UK had a 7-amino acid deletion at amino acid positions 2125–2131 in comparison with CSBV-GZ and CSBV-LN, and the CSBV-GZ and CSBV-LN had a 6-amino acid deletion at amino acid positions 2144–2149 in comparison with SBV-UK (Figure 7).

## 4. Discussion

**Figure 8:**
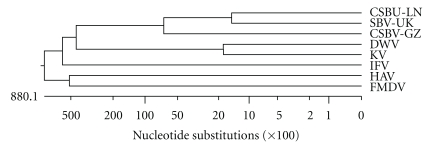
Phylogenetic analysis of RdRp amino acid sequence from CSBV-LN, CSBV-GZ, SBV-UK, DWV, KV, IFV, HAV, and FMDV. HAV and FMDV were used as an outgroup.

The nucleotide sequences (8863 nucleotides) contain a large ORF encoding 2848 amino acids. The genomic organization of CSBV clearly resembles that of typical Picornaviridae with structural proteins at the 5′end and the nonstructural proteins at the 3′end arranged in a similar order. DNASTAR software was used to carry out the phylogenetic analysis. Phylogenetic analysis of RdRp amino acid sequence shows that the CSBV-LN, CSBV-GZ, and SBV-UK belong to SBV which with DWV and KV tends to group together (Figure 8). The structural proteins precedence ordering of DWV has been determined, which is 5′-VP2 VP4 VP1 VP3-3′ [20]. So the similar structural proteins precedence ordering was predicted in CSBV, which is 5′-VP2 VP4 VP1 VP3-3′. The putative protease cleavage sites were predicted based on the size estimated of structural proteins by PAGE, and the precedence ordering of structural proteins was deduced using NetPicoRNA 1.0 Server from http://www.cbs.dtu.dk/services/NetPicoRNA/ [14]. The VP3 protease site (GAAQ**Q** ^1078^LTAS) conforms to the classic pattern for viral 3C proteases that cut after either glutamine (Q) or glutamic acid (E) [17, 21]. 

Using the positions of these residues as a guide, we found several putative recognition sites for the CSBV 3C protease in the polyprotein. All sites are suitably located for the separation of functional viral proteins from the polyprotein, with excellent agreement between the estimated and predicted molecular weights for the structural proteins. The most intriguing sites occur at amino acids 161 (KESI**Q** ^161^G**D**AT), 392 (VPLT**Q** ^392^M**D**VI), 428 (QSHN**Q** ^428^DKPK), and 743 (LPRVQ^743^MDTG). Site 161 could separate a leader polypeptide (L protein) from the polyprotein. Site 392 would, together with the VP1 N terminus, generate VP4, which is usually part of a precursor protein(VP0) together with VP1. Site 428 could separate VP1 from the polyprotein. Sit 1655 (FVTTQ^1655^GDFA) could separate the helicase from the polyprotein. Site 2308 (QPVVQ^2308^LEDW) is very similar to the confirmed VP3 protease site and would perfectly process the N terminus of the 3CD protein comprising the 3C protease and RNA-dependent RNA polymerase (RdRp) [22]. 

Analysis of the conserved protein domains and the proteolytic processing sites identified a leader protein (Lprotein) precedingVP2, followed by a putative VP4, VP1, and VP3, a helicase, VPg, 3C protease, and RdRp, all with recognizable and conserved protease sites in suitable locations for processing.

The emergence and reemergence of viral infectious diseases are often influenced by the genetics of viruses [21]. Extensive genetic variation has been observed among SBV isolates. In comparison with CSBV-GZ and SBV-UK, the CSBV-LN had a 13-amino acid deletion at amino acid positions 710–719 and 727–729, and in comparison with CSBV-GZ and CSBV-LN, the SBV-UK had a 7-amino acid deletion at amino acid positions 2125–2131, and, in comparison with SBV-UK, the CSBV-GZ and CSBV-LN had a 6-amino-acid deletion at amino acid positions 2144–2149. Besides, the second in-frame AUG codon position of CSBV-LN is different from SBV-UK, whose second in-frame AUG codon is at 197 [10]. The observed genetic heterogeneity of CSBV/SBV could lead to the selection of more virulent viruses and to the emergence or reemergence of new forms of CSBV/SBV. The genetic variation could show CSBV/SBV genotypes and contained several phylogenetic clusters. What was the reason of genetic variation of CSBV/SBV? It was probably because of different geographic origins, honeybee species, or others, which should be further investigated.

## Figures and Tables

**Table 1:** Synthetic oligonucleotides for amplification of CSBV genome.

Primers^a^	Sequence (5′ to 3′)	Nucleotide positions^b^
S1 F	5′-GACCCGTTTTCTTGTGAGTTTTAG-3′	41–64
S1 R	5′-GTGTAGCGTCCCCCTGAATAGAT-3′	611–633
S2 F	5′-TATTCAGGGGGACGCTACAC-3′	614–633
S2 R	5′-TATTCCATCGGGGTTATTTG-3′	1713–1732
S3 F	5′-GGAGACGCGCATGGTAAAGA-3′	1644–1663
S3 R	5′-GCGCGGTAAATAAACACTCG-3′	2365–2384
S4 F	5′-ATGGGGGTAAGGGACAATCTG-3′	2290–2310
S4 R	5′-TGCTCTAACCTCGCATCAAC-3′	3423–3442
S5 F	5′-TTACGGGAGCAGCACAACA-3′	3391–3409
S5 R	5′-ATTTCCGATTTACCGATACC-3′	4287–4306
S6 F	5′-CGGTGCGTTATGAACCTTTT-3′	4243–4262
S6 R	5′-AATGCGTAGATTGAGGTGCC-3′	5333–5352
S7 F	5′-GCGCAACTGGCACCTCAAT-3′	5325–5343
S7 R	5′-TTCCAAATATACTTCCCACTGC-3′	6249–6270
S8 F	5′-GTGACGGCAGTGGGAAGTAT-3′	6262–6243
S8 R	5′-GCAGCCTCCTCAGGTGTTAGT-3′	7454–7474
S9 F	5′-TTTGGTAGCGGGGTGTAAG-3′	7322–7340
S9 R	5′-CATTGCGTGGTATCATT-3′	8501–8517
S10 F	5′-TACGAATCGTGATTCGAT-3′	1–18

S10 R	5′-TAAACAAATCGGTATAAGAGTCC-3′	379–401

^a^F: forward; R: reverse.

^b^Nucleotide positions of S1–S9 primer pairs refer to the published CSBV sequence [7] (Genbank accession no. AF469603); nucleotide positions of S10 primer pairs refer to the published CSBV sequence [10] (Genbank accession no. AF092924.1).
